# Unlocking the Potential of Left Cardiac Sympathetic Denervation: A Scoping Review of a Promising Approach for Long QT Syndrome

**DOI:** 10.7759/cureus.47306

**Published:** 2023-10-19

**Authors:** Nidhi Dubey, Tyagi J Ubhadiya, Vasudha S Garg, Harsh Vadnagara, Mihir H Sojitra, Siddharth Kamal Gandhi, Priyansh Patel

**Affiliations:** 1 Department of Internal Medicine, Civil Hospital Ahmedabad, Ahmedabad, IND; 2 Department of Neurology, Civil Hospital Ahmedabad, Ahmedabad, IND; 3 Department of Internal Medicine, Shri M. P. Shah Government Medical College, Jamnagar, IND; 4 Department of Internal Medicine, Medical College Baroda, Vadodara, IND

**Keywords:** long qt syndrome, jervell and lange-nielsen syndrome, implantable cardioverter-defibrillator, arrhythmia, romano-ward syndrome, congenital long qt syndrome, left cardiac sympathetic denervation

## Abstract

Left cardiac sympathetic denervation (LCSD) has emerged as an alternative therapy for individuals diagnosed with long QT syndrome (LQTS), a genetic disorder characterized by abnormal electrical activity in the heart and sudden cardiac death (SCD). This review examines the history and rationale behind LCSD in LQTS treatment, as well as the procedure, its efficacy, and indications along with the adverse effects that may be associated with it. LQTS presents with prolonged QT intervals on an electrocardiogram and can manifest as seizures, fainting, and SCD. Beta-blockers are the primary treatment for LQTS but some patients do not respond well to these medications or experience side effects. Additionally, implantable cardioverter-defibrillators (ICDs) are not always effective in preventing arrhythmias and can lead to complications. LCSD might offer an alternative approach by disrupting sympathetic activity in the heart. In humans, LCSD reduces the release of norepinephrine, normalizes the QT interval, and decreases the likelihood of life-threatening heart rhythms. The procedure does not impair heart rate or cardiac function due to the compensatory effects of the right cardiac sympathetic nerves. The surgical procedure for LCSD involves the removal of the lower half of the stellate ganglion and thoracic ganglia. Complete denervation is essential for optimal outcomes, while incomplete procedures are considered unacceptable. Traditional and minimally invasive approaches, such as video-assisted thoracic surgery (VATS), are available, with VATS offering shorter hospital stays and fewer complications. In conclusion, LCSD provides a viable treatment option for individuals with LQTS who do not respond well to beta-blockers or require additional protection beyond medication or ICDs. Further research and clinical experience are needed to enhance its acceptance and implementation in routine practice.

## Introduction and background

In 1957, a family was observed where multiple children displayed a combination of congenital bilateral neural deafness and QT prolongation on an electrocardiogram (ECG). These individuals experienced recurring episodes of fainting (syncope) and sudden death, indicating a potential genetic disorder [1]. The family pattern suggested autosomal recessive inheritance and it was later named Jervell and Lange-Nielsen syndrome. A few years later, another familial disorder characterized by QT prolongation, but without deafness, was identified and termed Romano-Ward syndrome. This disorder was inherited through autosomal dominant inheritance and was much more common than its recessive counterpart [1]. These cases emphasized the hereditary nature of the condition, and further investigations revealed that malignant ventricular arrhythmias were responsible for the syncope and sudden death observed in these individuals. They were later grouped as a single disorder called long QT syndrome (LQTS) [1].

LQTS is a frequently occurring genetic cardiac channelopathy, affecting approximately one in every 2000 individuals [2,3]. This prevalence only applies to infants who have an unusually long corrected QT (QTc) interval and does not take into account the number of individuals who carry a genetic mutation associated with the condition but do not show any symptoms or have a normal QT interval [3]. The significant prevalence of this disease highlights its importance and underscores the need for additional attention and focus on addressing it. This genetic heart condition is a significant contributor to the occurrence of life-threatening ventricular arrhythmias and sudden cardiac death in young individuals who have structurally normal hearts [4]. The treatment of LQTS includes various approaches, starting with conservative measures like avoiding medications that prolong the QT interval. The initial treatment of choice is beta-adrenergic blockade, which has shown effectiveness in around 80% of patients [5]. However, for those patients who still experience syncope or cardiac arrest despite receiving beta-blockade, there is evidence suggesting that left cardiac sympathetic denervation (LCSD) is a highly effective therapeutic option [5]. Generally, appropriately treated patients can safely engage in most forms of exercise, and implantable cardioverter-defibrillator (ICD) therapy is typically reserved for individuals at the greatest risk [6].

LSCD involves the removal of the lower half of the stellate ganglion, and the second to fourth thoracic ganglia of the sympathetic chain [7]. The recent review of the rationale for LCSD emphasizes its significant ability to prevent fibrillation in the heart. This is attributed to several factors, including a significant decrease in the release of norepinephrine at the ventricular level, the absence of a phenomenon called post-denervation super-sensitivity, and no impact on heart rate [7,8]. Despite evidence of its long-term efficacy and safety, LSCD still appears to be underused in routine clinical practice [9]. In this literature review, we wish to study LCSD as an effective treatment option for patients with LQTS.

Methodology

We conducted a thorough search using PubMed Central, MEDLINE, and PubMed databases. The search strategy was designed based on the medical subject headings (MeSH) vocabulary ("Long QT Syndrome"[Mesh]) AND ("Denervation"[Mesh]). No specific time limit was imposed on the search. To ensure the relevance and quality of the studies, certain criteria were applied, leading to the exclusion of case reports, duplicates, and articles in languages other than English. During the screening process, titles and abstracts of articles were carefully reviewed to select studies for further full-text examination. Two independent authors were involved in the screening, and any disagreements were resolved through discussion among all the authors until a consensus was reached. Eventually, 24 studies were unanimously included in the review. Among these, nine were systematic reviews, eight were retrospective observational studies, two were editorial comments, and five were review articles.

## Review

History of LCSD

LCSD was initially utilized by physicians as an effective therapy for angina prior to the discovery of beta-blockers [9]. Amidst unforeseen twists and turns, accompanied by the rekindling of a neglected procedure, a remarkable innovation emerged as it found a new and unprecedented purpose when in the early 1970s, a patient with LQTS was successfully treated using LCSD [6,9-12]. Schwartz et al. conducted experiments on cats and demonstrated that stimulation of the left stellate ganglion (LSG) resulted in prolonged QT intervals and T wave alternans, which are distinctive patterns observed in the ECG of LQTS patients [11,12]. As a result, LCSD was performed on the second LQTS patient on March 25, 1973. Both procedures proved highly successful, with the patients remaining symptom-free for over 45 years [10,11]. Despite these positive outcomes, cardiologists were not particularly enthusiastic about this approach. Schwartz et al. remained the sole advocate of this procedure until the early 2000s when Ackerman et al. introduced thoracoscopic LCSD for their patients, leading to a minimally invasive approach to sympathetic denervation [10,11]. Two large multicenter reports also supported LCSD as a treatment option for LQTS [11]. However, it was only in 2017 that this approach was acknowledged [11]. Despite its recognition, LCSD still lacks popularity among cardiologists as a viable treatment choice for LQTS [9].

Rationale of LCSD in LQTS

LQTS is an inherited disorder that affects the depolarization and repolarization phases of the heart's electrical activity, as seen on an ECG [3]. It is commonly inherited in an autosomal dominant pattern (Romano-Ward syndrome and Timothy syndrome), although recessive patterns (Jervell and Lange-Nielsen syndrome) can also occur [3,13]. Around 15% of cases are due to spontaneous mutations [14]. Individuals with LQTS may experience symptoms such as fainting, seizures, aborted cardiac arrest, or sudden cardiac death, even without any prior health issues [3,8,10,13-18]. Approximately 10% of cases of sudden infant death syndrome (SIDS) involve mutations in genes associated with LQTS [17]. These symptoms are often triggered by increased sympathetic activity in long QT syndrome type 1 (LQT1) patients, by sudden noises in long QT syndrome type 2 (LQT2) patients, or can occur at rest or during sleep in long QT syndrome type 3 (LQT3) patients [3,13,15,17,18].

The main ventricular tachyarrhythmia in LQTS is called torsades de pointes (TdP), a unique form of ventricular tachycardia [3,13]. It is usually self-limiting, causing temporary loss of consciousness. However, it can sometimes progress to ventricular fibrillation (VFib), leading to cardiac arrest or sudden death [3]. LQTS can be diagnosed by observing a prolonged QT interval on an ECG [3,13,14,16,18]. T-wave alternans, which indicate electrical instability, can identify patients at higher risk [3,11-12,18], and the presence of notches on the T waves, which is typical for LQT2 [18]. Their presence in patients already receiving treatment indicates persistent high risk and requires a reassessment of therapy [18]. Sinus pauses, not related to normal sinus rhythm, are another warning sign, particularly in patients with SCN5A mutations [3]. The association of different subtypes of LQTS with specific gene mutations is shown in Table 1.

**Table 1:** Associations of different gene mutations with LQTS subtypes LQTS: Long QT syndrome. The information in Table 1 is adapted from Markiewicz-Łoskot et al. [13], which is an article published under open access.

Subtype	Gene mutation	Comments
LQTS1	KCNQ1 (K_v_LQT1)	LQTS1, LQTS2, LQTS3, LQTS4, LQTS5, and LQTS6 are all associated with phenotypes of Romano-Ward syndrome and Jervell and Lange-Nielsen syndrome
LQTS2	KCNH2 (HERG)	
LQTS3	SCN5A (Na_v_1.5)	
LQTS4	ANK2 (ANKB)	
LQTS5	KCNE1 (mink)	
LQTS6	KCNE2 (MiRP1)	
LQTS7	KCNJ2 (Kir2.1)	LQTS7 is associated with the phenotype of Andersen-Tawil syndrome
LQTS8	CACNA1C (Ca_v_1.2)	LQTS8, LQTS9, LQTS10, and LQTS11 are associated with phenotype of Timothy syndrome
LQTS9	CAV3	
LQTS10	SCN4B	
LQTS11	AKAP9	

A Schwartz score of 3.5 or higher suggests a high probability of LQTS [3,17,18]. In terms of risk factors, female gender, QTc of 500 ms or longer, LQT2 genotype, and frequency of cardiac events before age 18 are associated with an increased risk of experiencing cardiac events for ages between 18 and 40 years. After age 40, LQT3 genotype and recent syncope (within the past two years) are significant risk factors [13]. LQT3 patients have a higher lethality of cardiac events compared to LQT1 and LQT2 patients, indicating the need for more aggressive treatment in LQT3 cases [13]. QT interval duration is the most reliable predictor of cardiac events in LQT1 and LQT2 patients. A QTc exceeding 500 ms identifies patients at the highest risk of developing symptoms by age 40. Males with LQT3 may also be at higher risk, regardless of QT interval duration [13].

Beta-blockers are the preferred treatment for LQTS as they prevent the dispersion of repolarization caused by sympathetic stimulation, reducing the risk of ventricular arrhythmias [13]. They do not directly shorten the QT interval but rather minimize the triggered activity by modulating adrenergic activity in the heart [14]. In LQT1, beta-blockers are highly effective due to their impact on adrenergic stimulation [13,17]. However, noncompliance and the use of drugs that prolong the QT interval can still lead to life-threatening failures in LQT1 patients [3,8,18]. LQT2 patients still experience severe events despite beta blockers, but many of these can be successfully resuscitated [3,8,18].

In the context of LQT3 patients, there have been reports of a higher frequency of major events despite the use of beta-blockers [3,8,18,19]. Schwartz et al. argued that this misconception has led to the incorrect belief that beta-blockers have limited or no value in treating LQT3 patients. They attribute this misunderstanding to the inclusion of LQT3 patients who experience events during the first year of life, which is associated with a significantly poor prognosis regardless of treatment [3,8,18]. Contrarily, Schneider et al. counter this notion by suggesting that treatment of LQT3 with beta-blockers may have the potential to induce pro-arrhythmic effects due to their concurrent ability to slow down heart rate [19].

Patients with severe forms of LQTS, such as Jervell and Lange-Nielsen syndrome or Timothy syndrome, and patients who experience symptoms in the first year of life, also often require additional protection beyond beta-blockers [3,8,18]. One major concern with beta-blocker treatment is the possibility of bradycardia [3,18]. Many patients do not respond well to high doses of beta blockers or cannot tolerate them. Around 30% of patients do not show improvement with medical therapy and continue to experience symptoms [13,15,16,19]. Because of their side effects, some patients struggle with fatigue and a decrease in exercise capacity, which can significantly affect daily life or athletic activity, thus leading to poor compliance and patients looking for alternative treatment options [19]. Unfortunately, about 10% of patients may still face cardiac arrest or sudden cardiac death despite beta-blocker therapy [16].

ICDs do not prevent the occurrence of TdP, but they can effectively prevent sudden cardiac death when TdP persists or progresses into VFib [13]. Schwartz et al. suggested that ICD implantation should be considered in various scenarios - all patients who have survived a cardiac arrest while adhering to proper drug therapy; patients who have survived a cardiac arrest, excluding those with reversible or preventable causes, and possibly some individuals with previously undiagnosed and untreated LQT1; individuals with LQTS-triggered syncope despite optimal beta-blocker treatment, when the option of LCSD is unavailable or not chosen after discussions with the patient; patients with syncope despite full-dose beta-blockers and LCSD; exceptionally, asymptomatic post-pubertal LQT2 women with a QT interval equal to or greater than 550 ms, as well as asymptomatic patients with a QT interval above 550 ms who exhibit signs of high electrical instability or other indicators of high risk despite beta-blockade and LCSD (e.g., long sinus pauses followed by abnormal T-wave morphologies) [3,8,18]. Other experts also share similar criteria [14,17].

However, there is a significant discrepancy in the utilization of ICDs among patients with LQTS. Schwartz et al. highlight that some programs in the United States (US) implant ICDs in approximately 80% of patients with LQTS, while in two of the largest LQTS clinics worldwide (in Pavia and the Mayo Clinic), these rates are approximately 3% and 15%, respectively [8,18]. It is important to base current knowledge on the largest ICD study conducted thus far, which involved 233 LQTS patients. This study revealed a concerning finding, the majority of patients who received ICDs had not experienced a cardiac arrest, and many of them had not even failed beta-blocker therapy [8,18]. Schwartz et al. addressed this discrepancy by saying that for the clinical cardiologist, the choice to implant an ICD is relatively straightforward. If appropriate shocks occur, it can potentially save the patient's life. Even in the absence of shocks, although there may be complications, it is considered the best course of action for the patient's protection. However, deciding against ICD implantation could have legal implications if the outcome is tragic and there is not a valid justification supporting that decision [3,8,18].

ICDs are also not always effective in preventing ventricular arrhythmias. In some cases, they can inadvertently stimulate the release of norepinephrine, leading to episodes of severe electrical disturbances in the heart known as electrical storms. These storms can have serious consequences, causing significant illness, a decline in overall well-being, and even fatal outcomes, including device-related malfunctions, inappropriate therapies, infections, and psychological stresses [15,19,20]. Additional treatment options may be required for patients with highly arrhythmogenic conditions due to the limitations and side effects of drug-based therapy and the collective medical conditions accompanying the use of an ICD [20].

The balance between quality of life and clinical effectiveness appears to be a challenge, often seen as mutually exclusive in therapeutic approaches. However, this perspective is limited in scope. In this context, we will emphasize and explore an alternative approach that integrates treatment efficacy and quality of life, specifically, LCSD [10]. LCSD has emerged as a safe and supplementary treatment choice for individuals diagnosed with LQTS [20]. Despite the evident efficacy of LCSD in decreasing the occurrence of syncopal episodes and enhancing both short-term and long-term survival rates, its use has not garnered widespread acceptance or popularity [16].

Preclinical studies have provided evidence that stimulating the LSG, a part of the sympathetic nervous system, can increase the duration of the QT interval and create heterogeneity in repolarization. Stimulating the LSG can also induce T wave alternans, which is a characteristic change associated with LQTS [16]. On the other hand, blocking the LSG has been found to raise the threshold for VFib and prolong effective refractory periods, which led to the investigation of surgical denervation in LQTS patients [15,16]. Animal models mimicking LQTS have shown that isoproterenol, a medication that stimulates the sympathetic nervous system, can trigger ventricular arrhythmias [16]. In patients with LQTS, symptoms like near-fainting or fainting episodes are often triggered by sympathetic activation, typically caused by startling situations, fear, or physical exercise. Additionally, beta-blockers, which are medications that block the effects of adrenaline, have been found to prevent cardiac events, including torsade de pointes [16]. In humans, LCSD prevents the release of norepinephrine from the left-sided cardiac nerves into the ventricles of the heart [10,11,18,19]. It increases the VFib threshold making it more difficult for the heart to enter a fibrillation state [10,11]. It also brings the prolonged QT interval back to normal, decreases the variability in QT duration, and consequently lowers the likelihood of life-threatening abnormal heart rhythms [16]. It is important to note that LCSD does not decrease heart rate or cardiac contractility due to the compensatory effect of the right cardiac sympathetic nerves [8,10,11,18,19]. Due to the latter, LCSD does not impair left ventricular performance either [11]. LCSD causes denervation at the preganglionic level, which means that the connections between nerve cells (synapses) are removed and cannot be regenerated. This lack of reinnervation prevents the restoration of normal nerve function. In contrast, LCSD, unlike post-ganglionic denervation, does not lead to post-denervation super-sensitivity, which could potentially cause dangerous irregular heart rhythms [8,10,11,18]. Another significant difference between beta-blockers and LCSD is that the latter also prevents the increase in repolarization heterogeneity caused by alpha-adrenergic activation, specifically early and delayed after-depolarizations [11]. Inflammation has been observed in stellate ganglia removed from patients who underwent sympathectomy, indicating potential involvement of inflammation in these conditions [2,9].

Procedure

**Figure 1:**
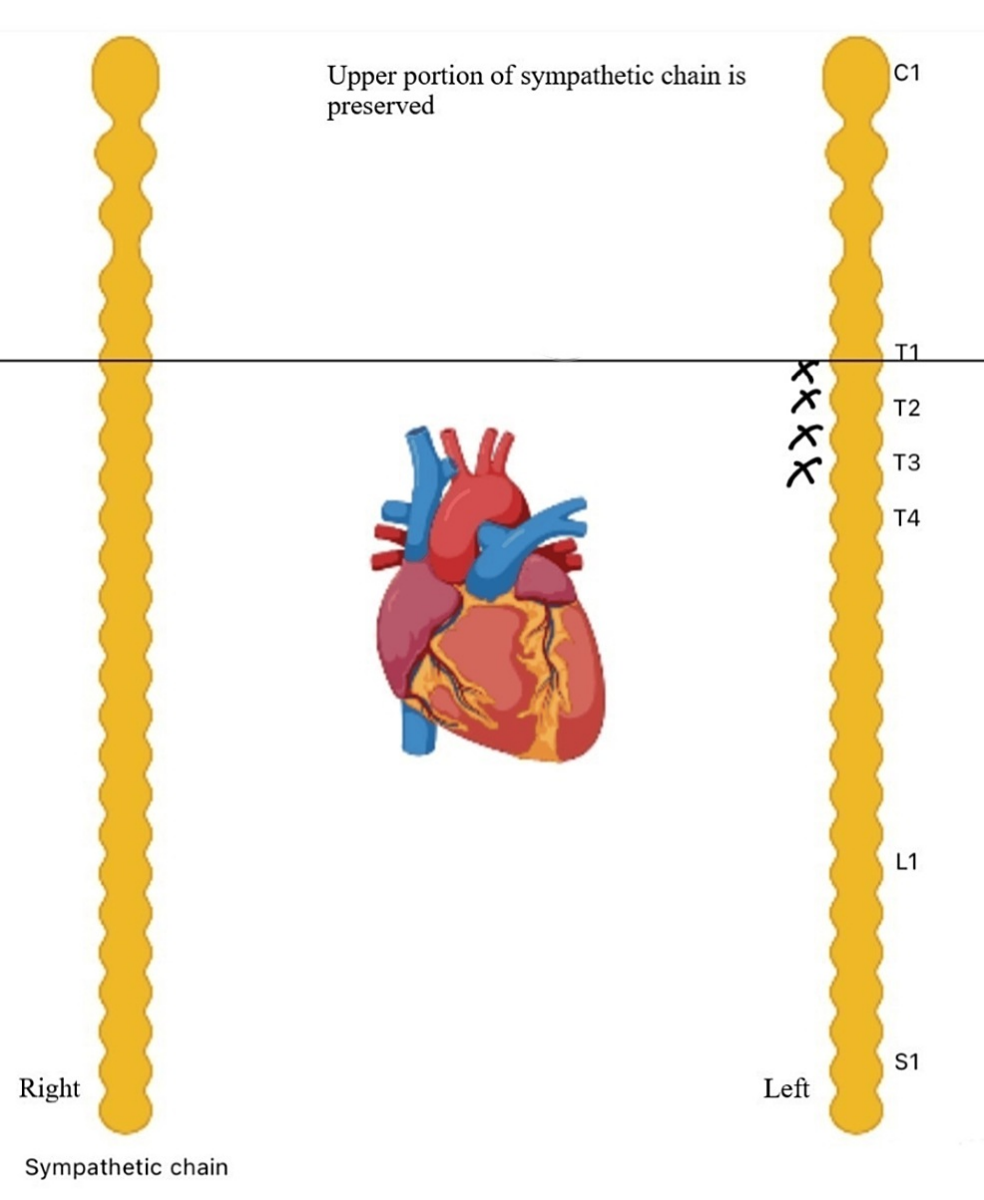
High thoracic left sympathectomy Image credits: Nidhi Dubey

The surgical procedure used in LCSD is known as high thoracic left sympathectomy (HTLS), which involves removing the lower half of the stellate ganglion (T1) and the thoracic ganglia from T2 to T4. It is important to preserve the upper portion of the LSG to avoid Horner's syndrome [3,8,10,12,14-16,18-21]. Previous studies have shown that incomplete denervation leads to higher failure rates, so leaving behind both stellate ganglia or only removing T4 is considered unacceptable and ethically concerning. These incomplete procedures should not be considered as proper HTLS surgery [10]. The ideal method for HTLS is an extrapleural approach, removing the necessity of a thoracotomy. For small infants or when local surgeons lack experience, Schwartz et al. recommend using the traditional and straightforward method, which involves making an incision in the third left intercostal space. This approach provides a clear view of the stellate ganglion and the sympathetic chain [3,8,18,19]. However, these surgical techniques can be extensive, requiring several days of hospitalization [16]. Video-assisted thoracic surgery (VATS) provides a minimally invasive alternative, which takes a brief span ranging from minutes to hours to complete and has no major complications. Patients can be discharged a few days after the surgery [12,16]. A study at the Mayo Clinic shows that there are no age restrictions for the VATS approach [8]. Figure 1 demonstrates the procedure for HTLS.

Studies

In 1995, a study involving a small group of 10 patients diagnosed with LQTS was conducted. The study found that after undergoing LCSD, the QTc interval decreased by an average of 30 ms. The patients were monitored for an average duration of 1.3 years, and all except one patient remained free of symptoms. Unfortunately, the patient who experienced symptoms after surgery passed away suddenly 10 months later [21]. In a preliminary study in 1991, a total of 85 patients were involved. The researchers observed a significant decrease in the occurrence of cardiac events, as the rates dropped substantially from 99% to 45% [15]. A comprehensive analysis was performed on a group of 123 patients who underwent LCSD after failing to respond to beta-blocker therapy. The findings of the study indicate a notable improvement in their condition. Specifically, the occurrence of cardiac events, particularly syncopal attacks, and cardiac arrests, decreased by 50% following LCSD [16]. Furthermore, the survival rates for these patients were quite promising, with a 94% survival rate after five years and a survival rate exceeding 80% after 10 years post-surgery [16].

In 2004, Schwartz et al. conducted a follow-up case series involving 147 LQTS patients who underwent LCSD within the previous 35 years. These patients were considered to be at exceptionally high risk, with 99% experiencing symptoms, an average prolonged QTc interval, previous cardiac arrest in 48% of cases, and recurrent syncope despite receiving a maximum dosage of β-blockers in 75% of cases. Over the course of an average eight-year follow-up, there was a substantial 91% reduction in cardiac events. LCSD was found to shorten the duration of QTc by an average of 39 ms, demonstrating its ability to modify both the trigger and the substrate (underlying electrical remodeling) [8,18,20,22]. In a study conducted in 2012, Schneider et al. examined a group of five patients diagnosed with LQTS. Four of these patients were identified as having mutations in the LQT1, LQT3, and LQT8 genes [19]. The decision to perform LCSD was based on the presence of severe symptoms that were not adequately controlled by medical therapy, including high-dose beta-blockers for all patients. The median duration of follow-up after the procedure was 1.6 years [19]. The findings of the study revealed that none of the patients demonstrated a significant reduction in the duration of the QTc interval following LCSD. However, all of them experienced the resolution of symptoms after undergoing the procedure [19].

In a retrospective study conducted by Hofferberth et al. in 2013, the effectiveness of LCSD was examined in patients with life-threatening ventricular arrhythmias. The study included 12 patients diagnosed with LQTS who underwent LCSD and were subsequently followed up for an average duration of 37.25 months. The findings revealed that out of these 12 patients, seven individuals achieved a state of being asymptomatic [12]. In a study, it was demonstrated that among 29 patients who had previously experienced symptoms, there was a reduction in cardiac events following LCSD. During a follow-up period of 3.6 years, 79% of these patients did not experience any further breakthrough cardiac events [15]. A study published in 2014 provided evidence that LCSD significantly reduces cardiac events in individuals with LQTS. The long-term survival without cardiac events was relatively high, with rates of 46% at five years and 59% at two years [17]. These studies show that LCSD is highly effective in LQTS. Two Jervell and Lange-Nielsen syndrome patients were identified in 2008, one of whom had symptom resolution after LCSD, while the other experienced one event within the 26-month follow-up period [20]. Additionally, two other case reports demonstrated a decrease in symptoms of the same syndrome following LCSD. One patient remained symptom-free after the surgery, while the other exhibited ventricular ectopy immediately after the procedure but did not experience it during the 18-month follow-up [20]. In a study conducted by Coleman et al. in 2013, five patients diagnosed with Jervell and Lange-Nielsen syndrome underwent VATS-LCSD. Among these patients, only one experienced symptoms after the procedure during a mean follow-up period of 1.8 years. Thus, proving its effectiveness in Jervell and Lange-Nielsen syndrome [20].

The utilization of VATS-LCSD has been significantly limited on a global scale, and there is a lack of clarity regarding its long-term effects. In light of this, Li et al. undertook an investigative effort to explore and shed light on this matter in their retrospective study published in 2018. They performed VATS-LCSD on eight Chinese patients diagnosed with LQTS. Post-surgery, there was a notable reduction in the average QTc after 24 hours, and further reduction after one week, with no significant change in heart rate. No deaths occurred during the follow-up period, and all patients experienced an improvement in their clinical symptoms. The annual event rate decreased after surgery, thereby showing that VATS-LCSD exerts a notable influence on cardiac electrophysiology, thereby offering a valuable therapeutic avenue for individuals with LQTS [15]. Coleman et al. and Hofferberth et al. also used VATS-LCSD in their study as mentioned before with promising results [12,20].

Wang highlighted that the clinical outcome of LCSD seems to rely on the extent of sympathetic denervation and the expertise of the operators. He says that a group from Italy, which has conducted a significant majority of LCSD procedures worldwide, has reported the most significant decrease in cardiac events and mortality following LCSD [16]. Earlier research conducted on canines has shown that undergoing left unilateral stellectomy leads to an elevation in heart rate during physical exertion, an effect attributed to reflex mechanisms that enhance sympathetic activity originating from the remaining intact ganglia on the opposite side [23]. Taking inspiration from the aforementioned findings in canine subjects, Anderson et al. embarked upon an endeavor to ascertain the veracity of such observations in human beings. In a retrospective analysis conducted by them between 2006 and 2017, 55 patients with LQTS who underwent LCSD were examined [23]. The findings of the study revealed that LCSD offers protection against LQTS-triggered events without posing any adverse effects on peak heart rate, cardiopulmonary fitness, or cardiac contractility, as assessed through treadmill exercise stress testing and echocardiography [23].

In the latest study published by Dusi et al. in 2021, based on their 50 years of experience performing LCSD on 125 LQTS patients, evidence of the long-term efficacy of LCSD was presented. The patients were retrospectively divided into four groups based on their clinical/genetic status as very high risk, aborted cardiac arrest (ACA), syncope and/or ICD shocks on beta-blockers, and primary prevention. Only 17% of patients in the very high-risk group showed no symptoms, whereas the percentages for the other three groups were significantly higher at 52%, 47%, and 97%, respectively. The mean yearly cardiac event rate was reduced by 86%. Among the 45 patients with syncope and/or ICD shocks before LCSD, none experienced ACA or sudden death as their first symptom after the procedure, and a post-LCSD QTc interval of <500 ms at six months predicted an excellent outcome. Individuals whose QTc interval was equal to or greater than 500 ms had a 50% probability of reducing it by an average of 60 ms. The results of LCSD were not influenced by common genotypes. The study also highlighted that the level of protection against arrhythmias was found to be directly associated with the extent of QTc interval reduction. It also gives evidence supporting the long-term safety and efficacy of LCSD in LQTS patients [9,11]. LCSD significantly enhances the quality of life for individuals, particularly those with LQTS who have an ICD implant. LCSD has a remarkable impact on reducing the frequency of ICD shocks, offering a substantial therapeutic benefit in mitigating the severe post-traumatic stress disorder that may arise from these shocks [3,8,10].

Complications

According to Schwartz et al., the untoward effects of LCSD are minimal and should not deter patients from considering it as an effective means to reduce the likelihood of VFib [18]. The unilateral effect includes the left hand becoming slightly warmer and dryer after the procedure, and in rare cases, this effect may extend to the left side of the forehead or foot [10,18,24]. One potential side effect that causes unwarranted concerns is the occurrence of Horner's syndrome, specifically left ptosis, which happens when the nerves passing through the stellate ganglion and directed to the left eye are dissected [10,18,20]. However, surgery preserving the upper half of the stellate ganglion can prevent Horner's syndrome. In their extensive experience with LCSD in hundreds of patients over 40 years, Schwartz et al. noted that Horner's syndrome occurred in 1.5% of cases. Nevertheless, the possibility that these nerves pass at a lower level within the stellate ganglion cannot be completely excluded, and Horner's syndrome may still occur in such cases [18,24].

During surgery, there may be temporary compression of the stellate ganglion, resulting in a slight lowering of the left eyelid. However, this is usually transient, lasting a few weeks. In some cases, the left eyelid may remain 1-2 mm lower, but this is typically not noticeable in normal social interactions [18,19,24]. The most concerning consequence of LCSD is neuropathic pain, which was rare with the previously used retro-pleural approach but is more likely to occur with the thoracoscopic approach due to the potential pulling of the sympathetic chain before its division. However, this pain is transient and typically resolves within the first few months after surgery [10]. Schwartz et al. recommended considering the administration of low-dose gabapentin starting 24 hours before surgery, particularly for patients at higher risk for this side effect, such as females aged 20-40 years with prior pain sensitization conditions [10].

In rare cases, patients may develop a transient harlequin appearance on the face after intense physical exercise or emotional excitement [10,24]. Waddell-Smith et al., in their retrospective study on the physical and psychological consequences of LCSD, reported that some patients experienced excessive sweating on the right side due to compensatory hyperhidrosis [24]. They also noted that a few patients reported difficulties in thermoregulation, experiencing a distinct hot and cold side of the body (right-left ratio of 50:50), making it challenging to regulate body temperature, especially in bed or in cold weather [24]. In this study, despite the occurrence of the side effects mentioned above and various others such as perspiration, paresthesia, and significant differences in hand temperature, the majority of patients expressed satisfaction with the LCSD procedure. They reported feeling positive and safer and were willing to recommend the procedure to others [24].

According to another study, which involved 100 patients with LQTS, the quality of life of the patients was assessed after undergoing LCSD. The results showed that the vast majority (92%) of patients and their families were satisfied with the surgery and would recommend it to other patients [10]. Furthermore, as mentioned before in the study by Anderson et al., LCSD does not have any negative impact on peak heart rate, cardiopulmonary fitness, or cardiac contractility [23].

Indications

In the past, guidelines did not strongly recommend the use of LCSD specifically for LQTS. However, the 2017 guidelines from the American Heart Association (AHA), American College of Cardiology (ACC), and Heart Rhythm Society (HRS) changed this perspective [10]. Based on extensive research on LCSD in LQTS, the AHA/ACC/HRS guidelines now provide a Class I recommendation for LCSD [7,10,23]. According to these guidelines, LCSD is recommended as a treatment option in two scenarios: first, for patients who experience a cardiac event despite being on pharmacotherapy or who have VFib-terminating shocks, and second, as a genuine alternative for patients who cannot tolerate or have contraindications to beta-blocker therapy [7,10,22].

If we think about its effectiveness among the different genotypes, a study by Schneider et al. shows that LCSD appears to be more effective in those with LQT1 and LQT3. LCSD, which does not decrease heart rate, may be particularly suitable for patients with LQT3 [19]. However, the results of LCSD were not affected by common genotypes in the study by Dusi et al. [11]. Earlier reports from 2004 already demonstrated the efficacy of LCSD as monotherapy in LQTS patients. In a study of 147 patients, 12% received LCSD as monotherapy, with 82% of them becoming asymptomatic and experiencing a significant reduction in QT interval. The use of LCSD as monotherapy has since increased, mainly due to beta-blocker intolerance [10]. While beta-blockers are considered the ideal therapy for LQTS, in cases of clear contraindications or true intolerance to beta-blockers, there is now sufficient evidence to support LCSD monotherapy, even for symptomatic patients. Close monitoring is advised for these patients, considering that the level of protection may be associated with the extent of QT interval shortening [10].

It is important to acknowledge that LCSD is not entirely effective in preventing cardiac events in high-risk patients. Therefore, the use of an ICD as a complementary treatment is recommended in such cases [15]. LCSD and ICD are not mutually exclusive and can complement each other by reducing the number of cardiac events and the risk of SCD [15]. The AHA/ACC/HRS guidelines emphasize that clinicians should consider modification of drug therapy; LCSD or ICD therapy as equally reasonable options for treatment intensification. They no longer mandate that LCSD should be the second-line choice after ICD [10]. This balanced approach counters the tendency seen in some regions to reflexively opt for ICD placement. The recent 2021 PACES Expert Consensus statement echoes similar sentiments [10]. For LQTS patients who are not fully protected by beta-blockers, it is crucial that they and their families are informed about LCSD and its additional protective benefits with fewer adverse events compared to ICDs. Physicians have a responsibility to provide adequate and unbiased information to patients, as failing to do so could lead to legal consequences [10]. It is disheartening to observe that many cardiologists worldwide, even in advanced countries, readily recommend ICD implantation for numerous LQTS patients without presenting them with the alternative of LCSD [10]. Merely having the ability to implant an ICD should not overshadow the need to fully inform families about available treatment options [10].

Limitations

Several limitations exist regarding the assessment and understanding of LCSD as a treatment for LQTS. Firstly, the majority of available studies investigating LCSD for LQTS are retrospective or observational in nature, lacking large-scale randomized controlled trials (RCTs). This absence of RCTs limits the ability to establish a cause-and-effect relationship between LCSD and clinical outcomes, hindering comparisons with other treatment modalities and the determination of long-term efficacy. Furthermore, the heterogeneity of study populations poses a challenge in assessing the generalizability of LCSD findings. LQTS is a rare condition, and studies often include small sample sizes, making it difficult to extrapolate the results to a broader population. Additionally, variations in patient characteristics, such as age, genotype, and disease severity, may influence the outcomes of LCSD. Thus, more research involving larger and more diverse cohorts is needed to gain a better understanding of the potential benefits of LCSD in different LQTS subtypes and patient profiles. Another limitation arises from the variability in surgical techniques employed for LCSD across studies. Factors such as the extent and location of the denervation procedure can vary, introducing inconsistency in outcomes. The lack of standardized protocols for LCSD hinders the ability to compare results across studies and draw definitive conclusions regarding its efficacy and safety. The establishment of consensus guidelines and standardized techniques for LCSD would help address this limitation and facilitate more robust research in the future. Short-term follow-up periods in existing studies represent an additional limitation. Many investigations on LCSD for LQTS have relatively short follow-up durations, typically ranging from a few months to a couple of years. Such limited follow-up may not capture long-term outcomes, including the recurrence of arrhythmias or the need for additional interventions. Longer follow-up periods are required to appraise the efficacy of treatment effects and evaluate the influence of LCSD on patient outcomes over time. Moreover, the current literature primarily focuses on LCSD in adult patients, while data on the pediatric population with LQTS is scarce. Given that LQTS commonly manifests in childhood, it is essential to determine the safety and efficacy of LCSD in this specific patient subset. Finally, the lack of comparative studies between LCSD and alternative treatment strategies is noteworthy. While LCSD is often considered for LQTS patients who are refractory to or intolerant of beta-blockers, direct comparative studies with other therapeutic interventions, such as different beta-blocker regimens or other surgical procedures, are lacking. Conducting comparative studies would provide valuable insights into the relative efficacy and safety of LCSD in the management of LQTS. Acknowledging these limitations is crucial for future research endeavors to obtain a more comprehensive understanding of LCSD as a therapeutic option for LQTS.

## Conclusions

In conclusion, LCSD is a promising surgical treatment for LQTS. It serves as an alternative option for LQTS patients unresponsive to beta-blockers or experiencing complications with ICDs. Numerous studies have demonstrated the effectiveness of LCSD in reducing syncopal episodes and improving short-term and long-term survival rates. Its adverse effects are minimal compared to the protection it offers. However, despite its efficacy and acceptance into standard guidelines as class 1 recommendations for LQTS, it is important to acknowledge that LCSD is not widely accepted or popular among cardiologists as a treatment choice for LQTS. To advance the field, further research is encouraged. Well-designed RCTs adhering to standardized protocols and incorporating long-term follow-up are essential to assess the durability of LCSD's benefits. Systematic reviews and meta-analyses should be encouraged to consolidate existing evidence, identify knowledge gaps, and provide a comprehensive overview of LCSD's potential as a transformative therapy for LQTS. Clinical cardiologists should consider adopting LCSD as a viable therapeutic option in their routine practice. By choosing LCSD over ICDs, which can adversely affect patients' quality of life, clinicians have the potential to improve patient outcomes and overall well-being. In conclusion, LCSD represents an exciting frontier in the management of LQTS. Integrating LCSD into routine clinical practice promises to significantly improve the lives of individuals with LQTS, reduce syncope episodes, and enhance overall patient well-being and quality of life.
